# Supplementary figures and images for: RAD51 is a druggable target that sustains replication fork progression upon DNA replication stress
Source: PLoS One. 2022 Aug 15;17(8):e0266645. doi: 10.1371/journal.pone.0266645 (PMC9377619; doi:10.1371/journal.pone.0266645)

S1 Fig.

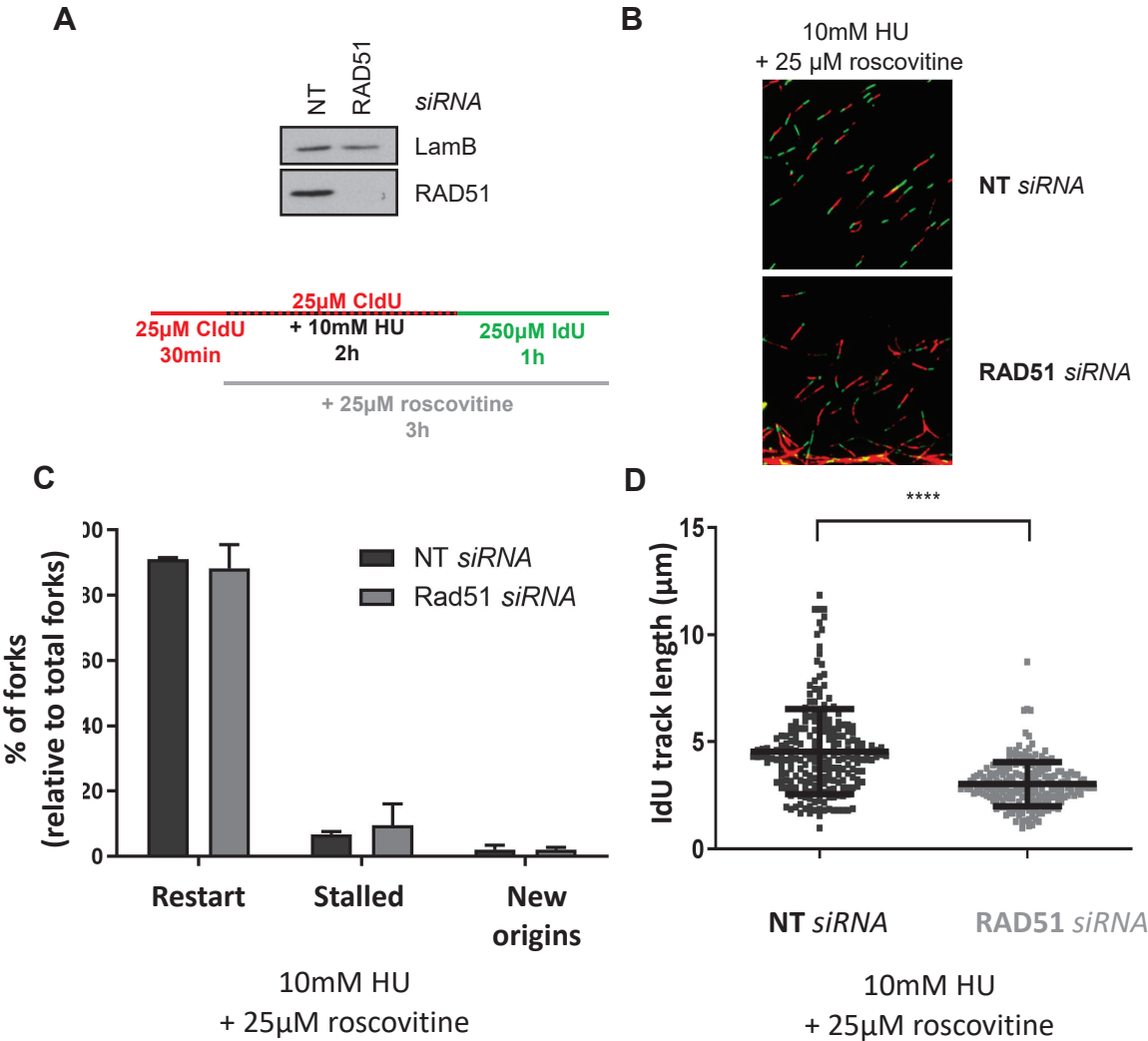

Supplement: S1 Fig — (A) Cells were transfected with the indicated siRNA (NT: non-target) and 48 hours later cells were harvested for WB analysis with RAD51. Lamin B (LamB) was used as a loading control (upper panel). hTERT-RPE transfected cells were labelled as indicated (bottom panel). After labelling, cells were harvested and prepared for DNA fiber analysis. (B) Representative DNA fiber images are shown. (C) At least 200 fibers of each condition in each experiment were used to calculate the percentage of restart, stalled forks and new origin firing events relative to total forks. Means and standard deviation (bars) of three experiments are shown. The statistical analysis was performed just in HU-treated cells (paired t-test, non-statistically significant differences were found). (D) DNA fibers from were used to measure IdU track length (second analogue). At least 200 fibers of each condition in each experiment were measured. One representative experiment out of three is shown (bottom-left panel, Mann-Whitney test, **** P value < 0.0001). See data used for quantification in S1 File. (PDF) [file pone.0266645.s001.pdf]

S2 Fig.

**A**

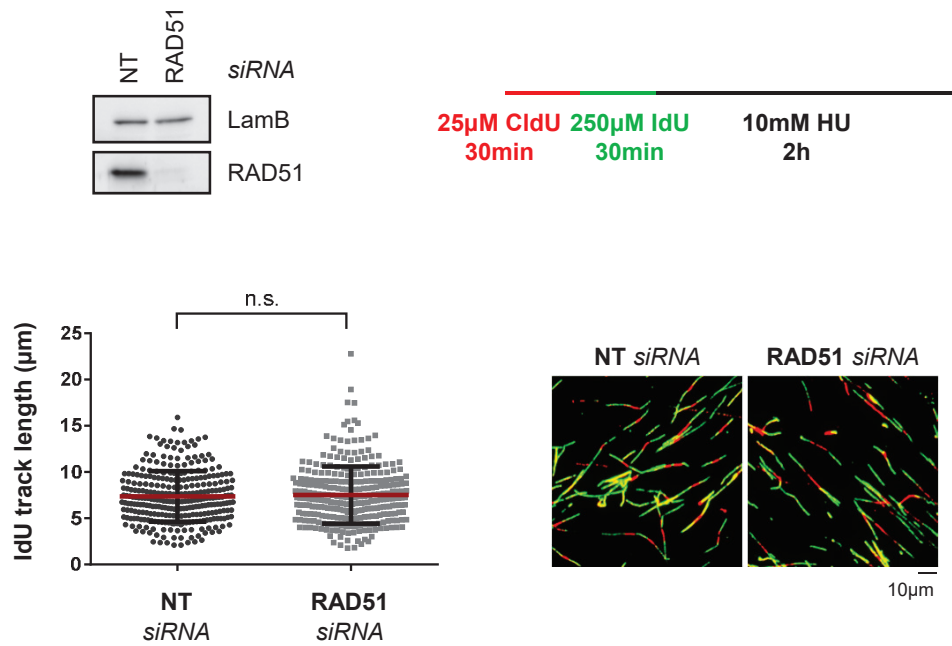

**B**

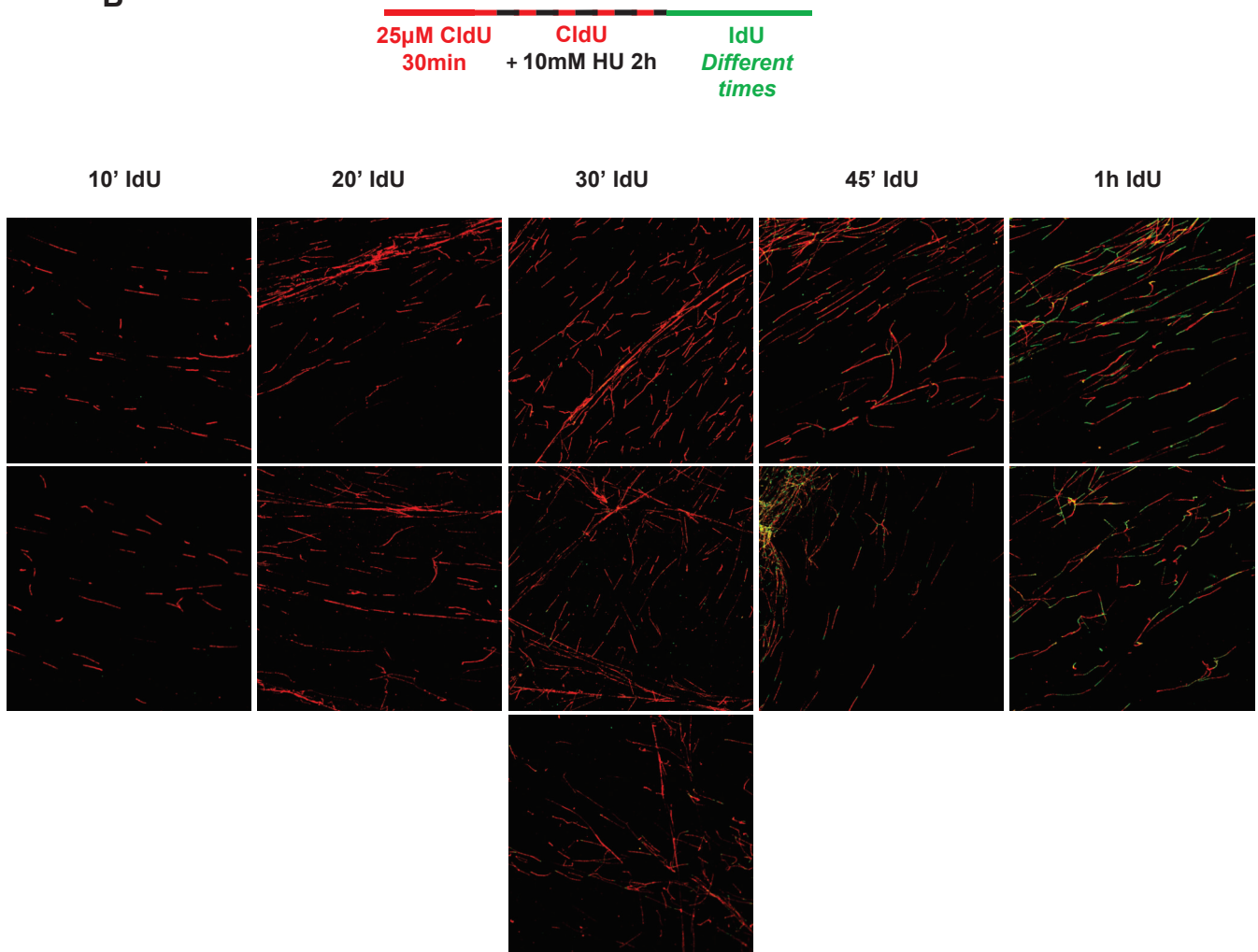

Supplement: S2 Fig — (A) RAD51 depletion does not cause fork degradation after an acute replication stress in hTERT-RPE cells. (B) Analysis of nucleotide incorporation upon 10mM HU treatment. A) hTERT-RPE cells were transfected with the indicated siRNA (NT: non-target) and 48 hours later cells were harvested for WB analysis with RAD51 antibody. Lamin B (LamB) was used as a loading control (upper-left panel). hTERT-RPE transfected cells were labelled as indicated (upper-right panel). After labelling, cells were harvested and prepared for DNA fiber analysis. Representative images are shown (bottom-right panels). The IdU track length was measured. At least 300 fibers of each condition in each experiment were measured. One representative experiment out of three is shown (bottom-left panel, Mann-Whitney test, n.s.: non-statistically significant). B) hTERT-RPE cells were incubated 30 min with CldU and then treated with 10 mM HU for 2h. After that, cells were washed and IdU was added to new fresh media. Cells were harvested at the indicated times and processed for fiber analysis. Duplicated or triplicated images are shown for different times. IdU incorporation was not detected until 30 min upon HU release. See data used for quantification in S1 File. (PDF) [file pone.0266645.s002.pdf]

**S3 Fig.**

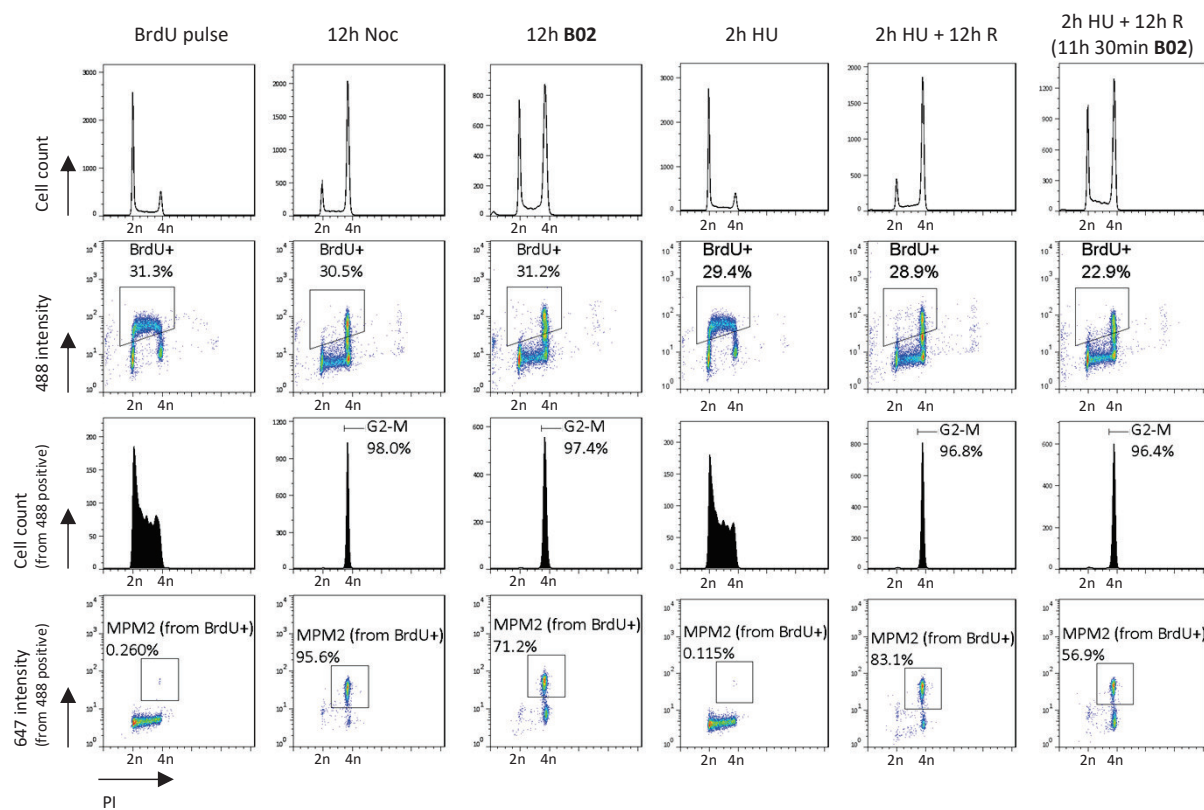

Supplement: S3 Fig — hTERT-RPE cells were labelled with BrdU and then treated during 2 hours with 10mM HU or left untreated for 12 hours into nocodazole-containing fresh medium, without (12h Noc) or with RAD51 inhibitor (12h B02) (upper panel). After HU treatment, cells were released into nocodazole-containing fresh medium, without (12h Noc) or with RAD51 inhibitor, added after 30 minutes of HU release (11h 30min B02). Flow cytometry analysis of approximately 15000 cells was performed to analyze the S-phase population, initially labelled with BrdU analogue (BrdU-488 positive cells). Cell cycle progression was analyzed by measuring mitotic cells (MPM2-647 positive from BrdU-488-positive population) relative to cells into G2-M phases (obtained by black DNA profiles from BrdU-488-positive population). Related to Fig 4. (PDF) [file pone.0266645.s003.pdf]

S4 Fig.

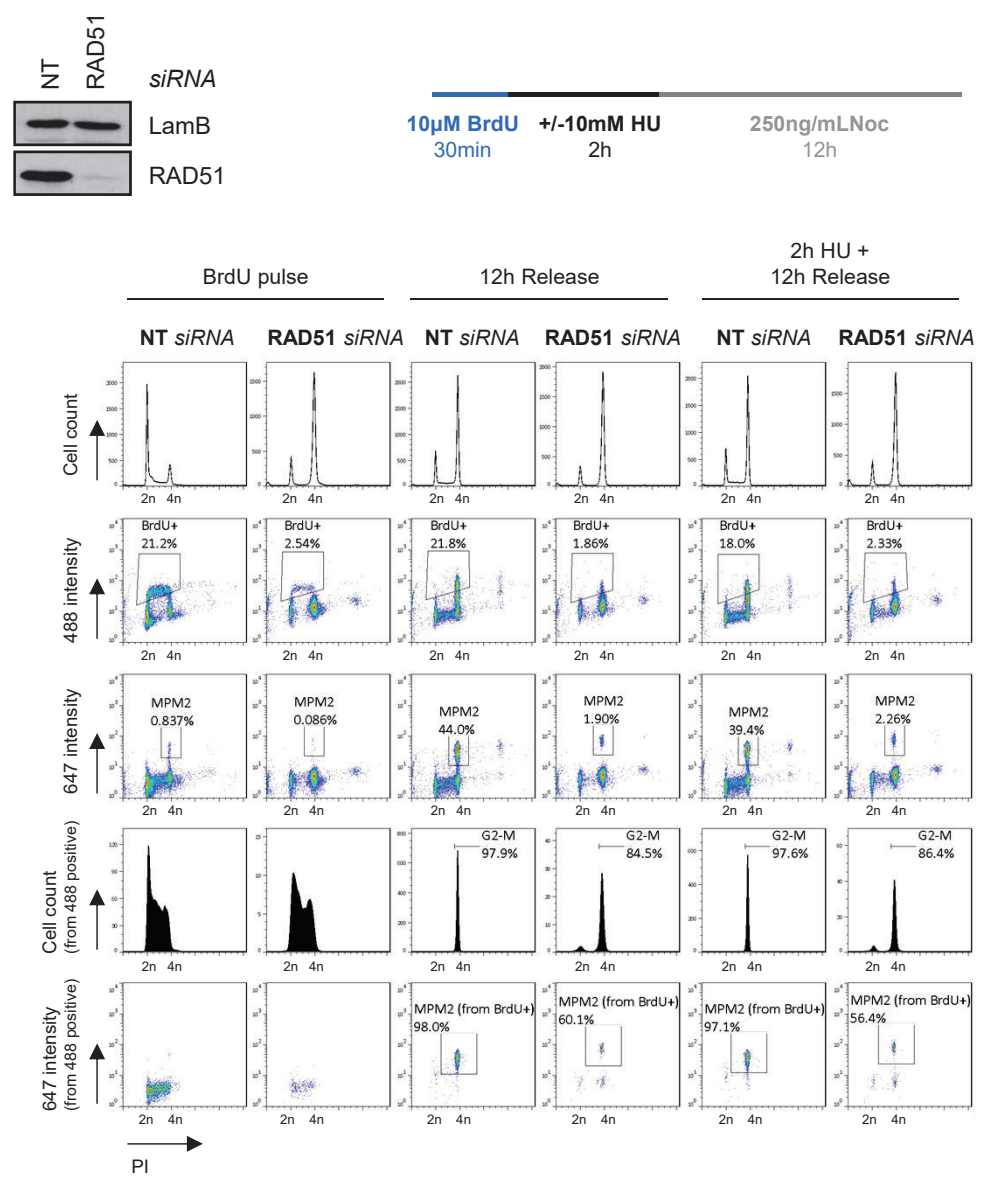

Supplement: S4 Fig — hTERT-RPE cells were transfected with the indicated siRNA (NT: non-target) and 48h later cells were labelled with BrdU and then treated with 10mM HU or left untreated (12h release) into nocodazole-containing media for 12 hours. HU-treated cells were then released into nocodazole-containing fresh medium for 12 hours (2h HU + 12h release). Flow cytometry analysis of approximately 15000 cells was performed to analyse the S-phase arrested (BrdU-488 positive) cells after HU treatment, and the recovery from this stress measuring mitotic (MPM2-647 positive) cells from BrdU positive population. (PDF) [file pone.0266645.s004.pdf]

S5 Fig.

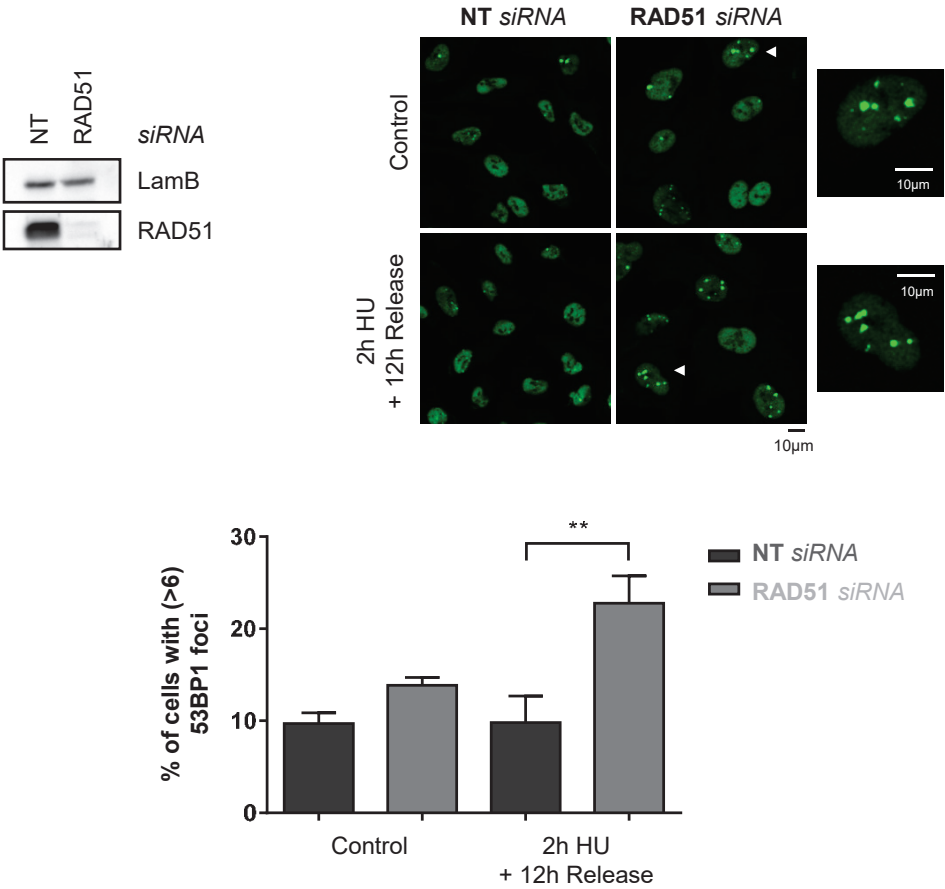

Supplement: S5 Fig — hTERT-RPE cells were transfected with the indicated siRNA (NT: non-target) and 48 hours later cells were treated with 10mM HU for 2 hours or left untreated for 12 hours (Control). After HU treatment, cells were released into fresh medium for 12 hours. Finally, 53BP1 immunofluorescence was performed. The control for KD was shown (upper-left panel). Representative images from each condition are shown (upper-right panels). Two cells with more than six 53BP1 foci, indicated with a white arrowhead in the representative images from RAD51-depleted population, are shown in more detail (upper-right panels). At least 500 cells were counted for NT-depleted cells and 200 cells were counted for RAD51-depleted cells in each experiment. Means and standard deviation (bars) of percentage of cells presenting more than six 53BP1 foci of two experiments in control and three experiments in HU conditions are shown (bottom panel). The statistical analysis was performed just in HU-treated cells (unpaired t-test, ** P value < 0.01). See data used for quantification in S1 File. (PDF) [file pone.0266645.s005.pdf]

**S6 Fig.**

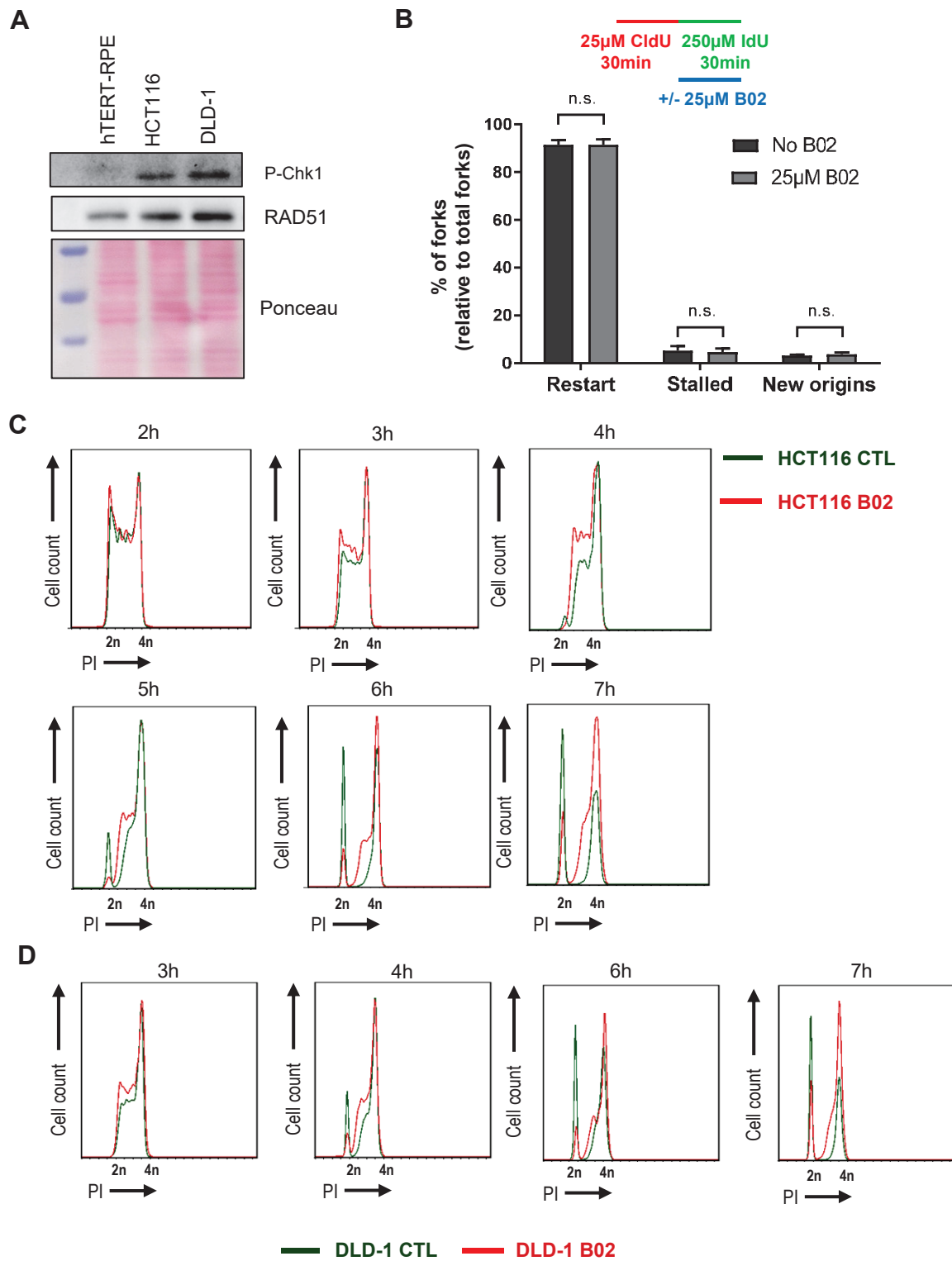

Supplement: S6 Fig — (A) Cells were lysed and P-Chk1 (Ser296) and RAD51 were analyzed by western blot. Ponceau was used as loading control (B) HTC116 cells were labelled as indicated in the upper-panel, adding the B02 inhibitor with the second analogue. After labelling, cells were harvested and prepared for DNA fiber analysis. Representative images are shown in Fig 6. DNA fibers were used to calculate the percentage of restart, stalled forks and new origin firing events relative to total forks. Around 1500 fibers from three independent experiments were counted in each condition. The average of those experiments is shown. Error bars represent standard deviation (paired t-test, n.s.: non-statistically significant). Related to Fig 6. (C) HCT116 cells were pulse labelled with BrdU and allow to proceed cell cycle in the absence of any drug (CTL, green profile) or in the presence of B02 (B02, red profile). Cells were harvested at the indicated times, fixed, permeabilized, and stained with propidium Iodide (PI) and with anti-BrdU antibody under denaturing conditions. DNA content (PI) of BrdU positive cells is shown. (D) as in (C) but with DLD-1 cells. See data used for quantification in S1 File. (PDF) [file pone.0266645.s006.pdf]

**S7 Fig.**

**A**

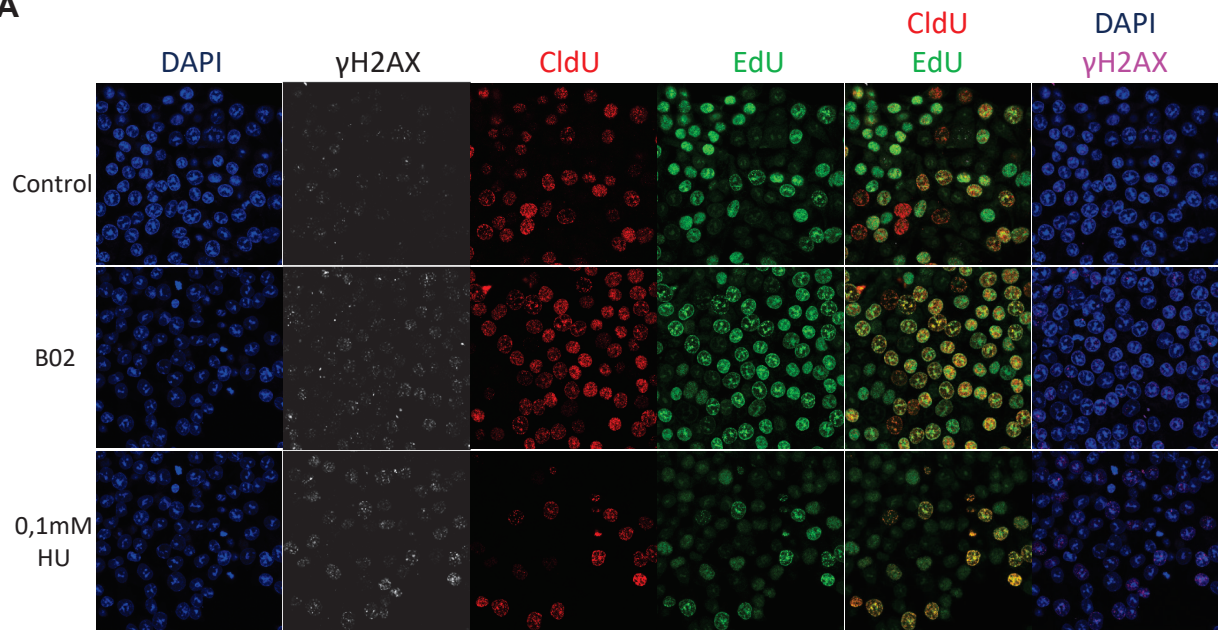

**B**

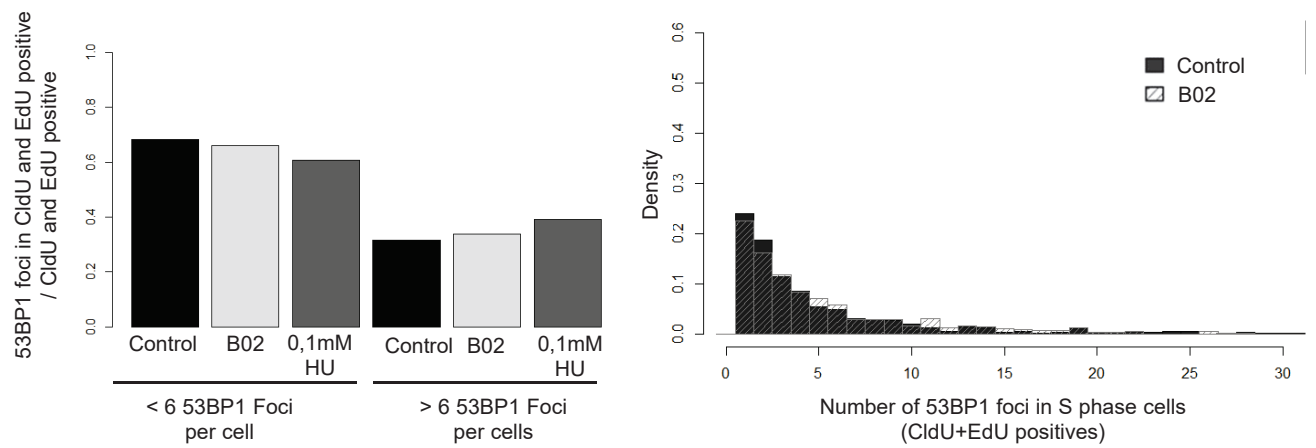

Supplement: S7 Fig — (A) Representative images of γH2AX, CldU and EdU immunofluorescence used for the quantification in Fig 6C are shown. (B) Cells were treated as in Fig 6, but 53BP1 instead of γH2AX was analyzed by immunofluorescence. Related to Fig 6. See data used for quantification in S1 File. (PDF) [file pone.0266645.s007.pdf]
