# Supplementary material for: RAD51 is a druggable target that sustains replication fork progression upon DNA replication stress
Source: PLoS One. 2022 Aug 15;17(8):e0266645. doi: 10.1371/journal.pone.0266645 (PMC9377619; doi:10.1371/journal.pone.0266645)

Figure 5A

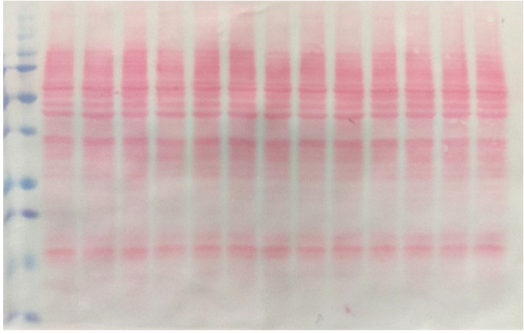

Ponceau gel 0.1mM HU

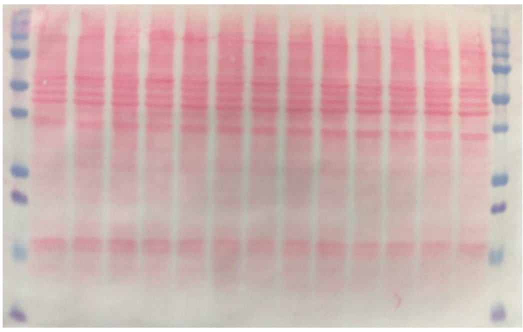

Ponceau gel 1mM HU

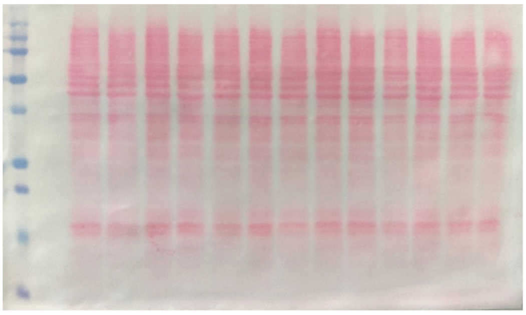

Ponceau gel 10mM HU

Figure 5A

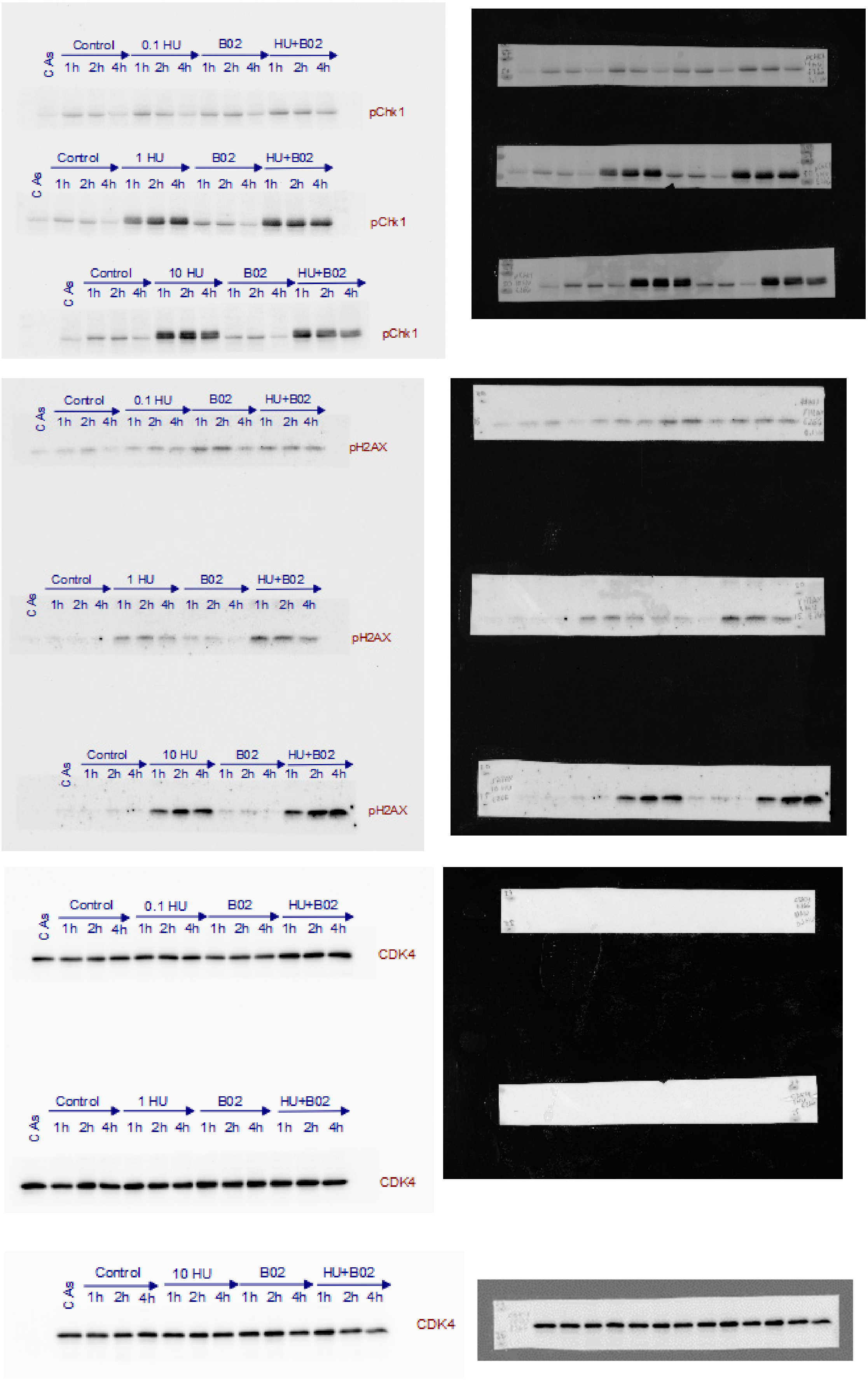

Control
0.1 HU
B02
HU+B02
C As
1h 2h 4h 1h 2h 4h 1h 2h 4h 1h 2h 4h
pChk1
Control
1 HU
B02
HU+B02
C As
1h 2h 4h 1h 2h 4h 1h 2h 4h 1h 2h 4h
pChk1
Control
10 HU
B02
HU+B02
C As
1h 2h 4h 1h 2h 4h 1h 2h 4h 1h 2h 4h
pChk1
Control
0.1 HU
B02
HU+B02
C As
1h 2h 4h 1h 2h 4h 1h 2h 4h 1h 2h 4h
pH2AX
Control
1 HU
B02
HU+B02
C As
1h 2h 4h 1h 2h 4h 1h 2h 4h 1h 2h 4h
pH2AX
Control
10 HU
B02
HU+B02
C As
1h 2h 4h 1h 2h 4h 1h 2h 4h 1h 2h 4h
pH2AX
Control
0.1 HU
B02
HU+B02
C As
1h 2h 4h 1h 2h 4h 1h 2h 4h 1h 2h 4h
CDK4
Control
1 HU
B02
HU+B02
C As
1h 2h 4h 1h 2h 4h 1h 2h 4h 1h 2h 4h
CDK4
Control
10 HU
B02
HU+B02
C As
1h 2h 4h 1h 2h 4h 1h 2h 4h 1h 2h 4h
CDK4

Figure 6B

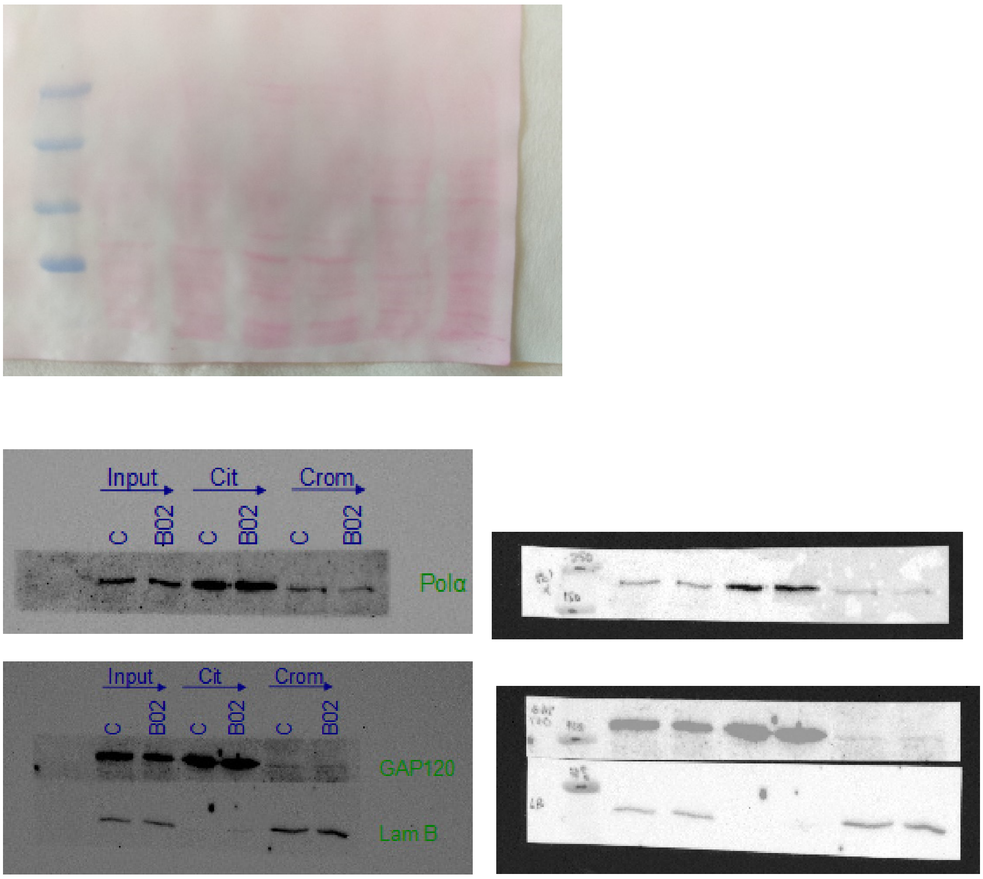
Input
Cit
Crom
C
B02
C
B02
C
B02
Polα
Input
Cit
Crom
C
B02
C
B02
C
B02
GAP120
Lam B

Figure S1

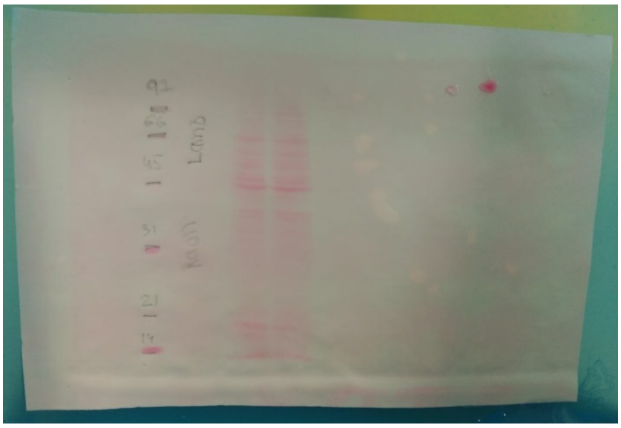

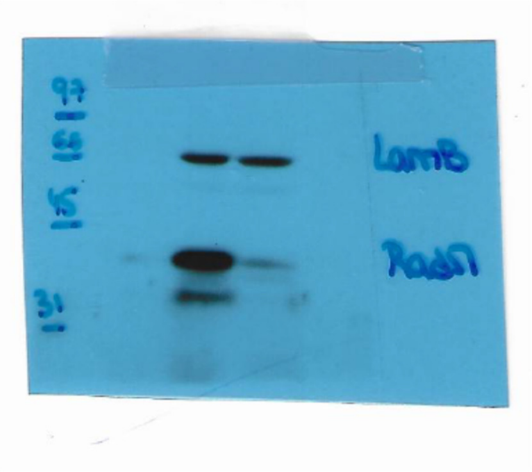

Figure S2

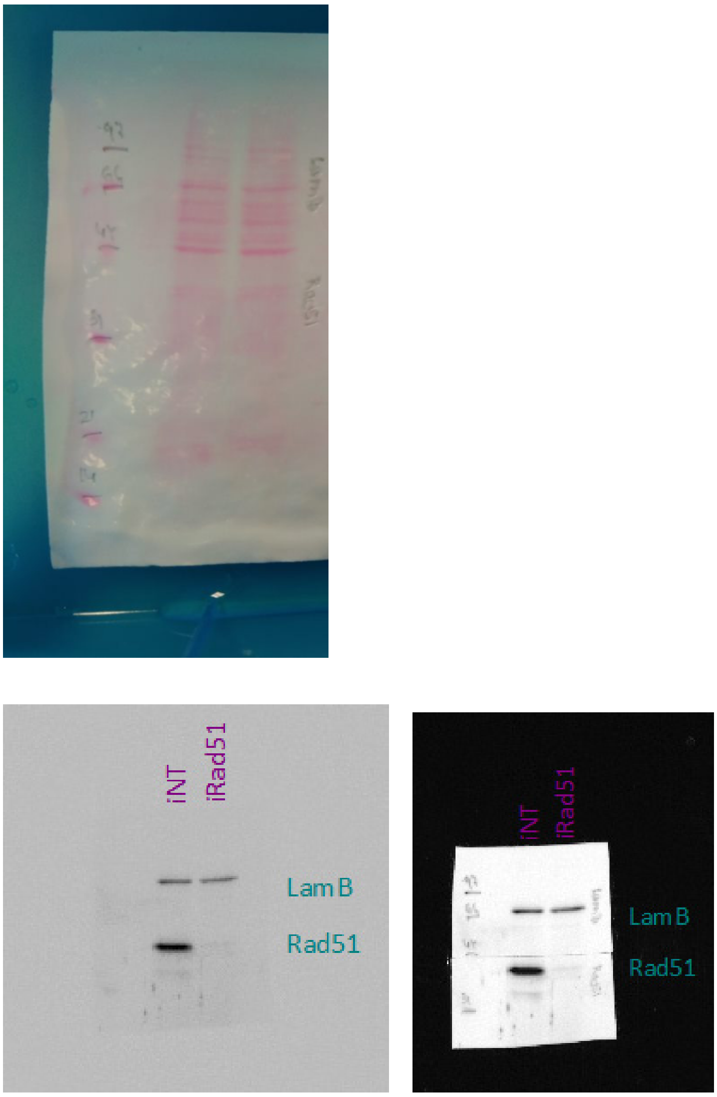

Figure S4

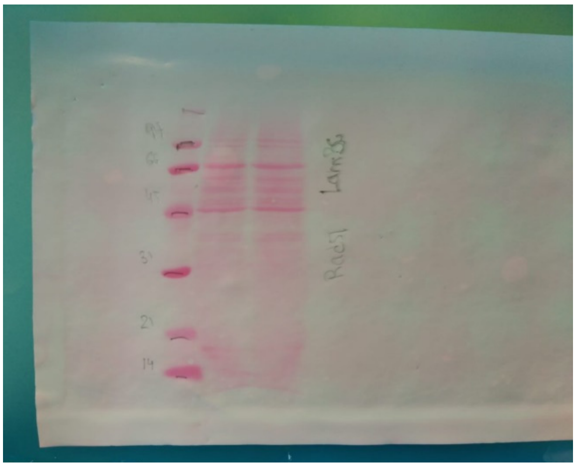

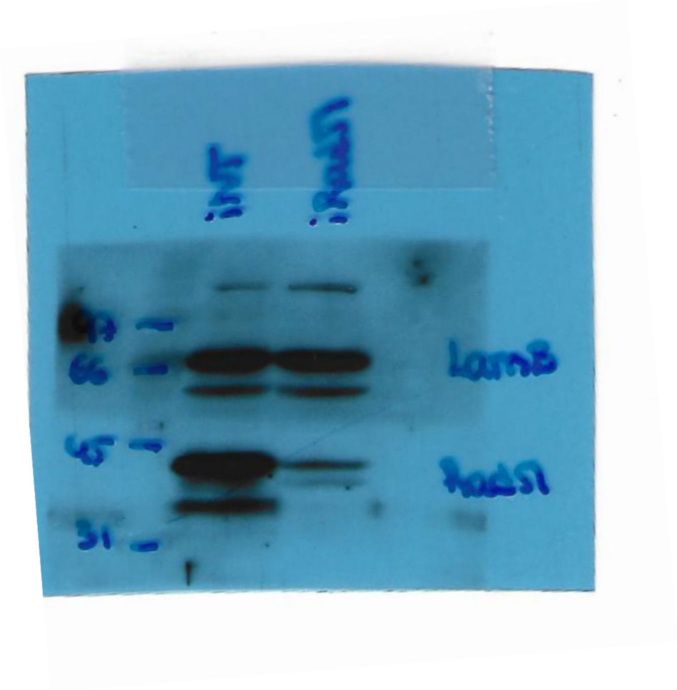

Figure S5

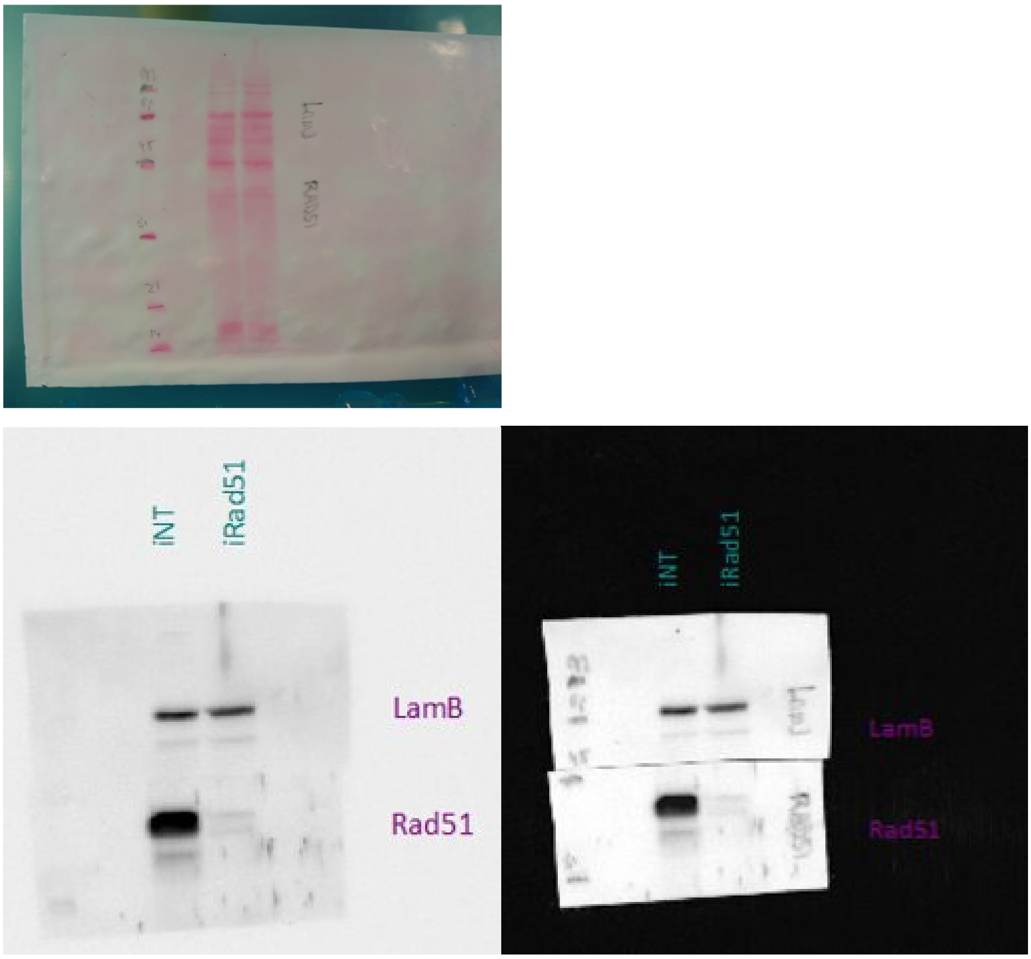

Figure S6

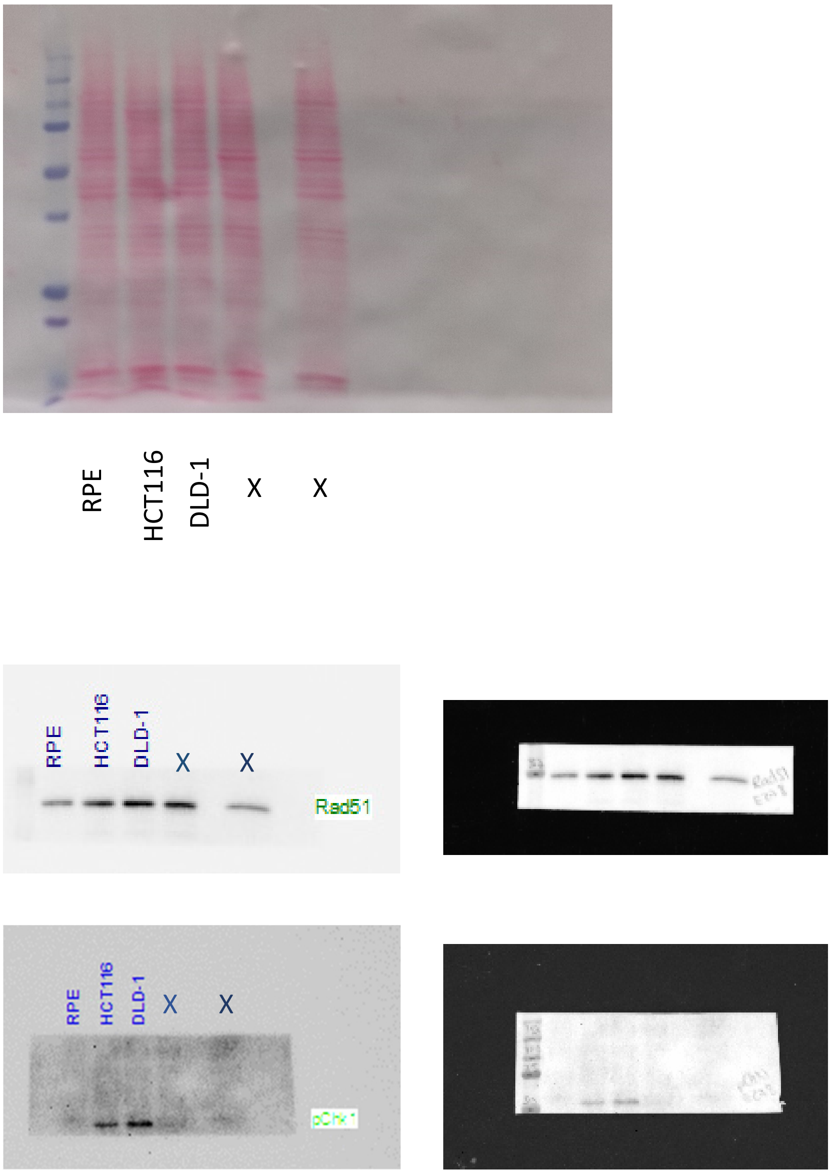

Supplement: S1 Raw images — (PDF) [file pone.0266645.s008.pdf]
